# Sensory organization of postural control after long term space flight

**DOI:** 10.3389/fncir.2023.1135434

**Published:** 2023-04-17

**Authors:** Nikita Shishkin, Vladimir Kitov, Dimitry Sayenko, Elena Tomilovskaya

**Affiliations:** ^1^Laboratory of Gravitational Physiology of the Sensorimotor System, Institute of Biomedical Problems of the Russian Academy of Sciences, Moscow, Russia; ^2^Department of Neurosurgery, Center for Neuroregeneration, Houston Methodist Research Institute, Houston, TX, United States

**Keywords:** computerized dynamic posturography (CDP), joint coordination, sensory organization test, microgravity, postural balance, biomechanics, sensory reweighting, postural control strategy

## Abstract

**Background:**

Alterations in motor control systems is an inevitable consequence of space flights of any duration. After the flight, the crew-members have significant difficulties with maintaining upright balance and locomotion, which last several days following landing. At the same time, the specific mechanisms of these effects remain unclear.

**Objectives:**

The aim of the study was to assess effects of long-term space flight on postural control and to define the changes of sensory organization caused by microgravity.

**Methods:**

33 cosmonauts of Russian Space Agency, the members of International Space Station (ISS) flights of duration between 166 and 196 days took part in this study. Computerized Dynamic Posturography (CDP) tests, which include assessment of visual, proprioceptive and vestibular function in postural stability, was performed twice before the flight and on the 3rd, 7th, and 10th days after landing. The video analysis of ankle and hip joints fluctuations was performed to investigate the basis of postural changes.

**Results:**

Exposure to long-term space flight was followed by considerable changes of postural stability (−27% of Equilibrium Score value in the most complicated test, SOT5m). Changes in postural strategies to maintain balance were observed in the tests which provide the challenge for vestibular system. In particular, increased hip joint involvement (+100% in median value and +135% in 3rd quartile of hip angle fluctuation RMS in SOT5m) into postural control process was revealed.

**Conclusion:**

Decrease of postural stability after long-term space flight was associated with alterations in vestibular system and biomechanically was revealed by increased hip strategy which is less accurate, but simpler in terms of the central control.

## 1. Introduction

Postural stability deficit is an inevitable consequence of space flights (SF) of any duration (Kozlovskaya et al., 1981; Paloski et al., 1992; Black et al., 1995; Reschke et al., 1998, 2009; Wood et al., 2015). It is well known that the visual, vestibular and proprioceptive sensory systems are all critical and involved in control of the postural balance in humans (Nashner, 1971). In 1970, Nashner introduced a method termed Computerized Dynamic Posturography (CDP), which development described in Black and Paloski (1998) and proposed this approach to investigate the contributions of different sensory inputs using Sensory Organization Tests (SOT). These tests are based on creating conditions when information from either visual or proprioceptive inputs becomes unreliable or insufficient to determine the orientation of the body in relation to the gravitational vertical. The tests include tilting of the support surface and the visual environment together with the inclination of the body. It is noteworthy that, according to previous studies (Nashner, 1971) vestibular afferentation is not decisive in determining the degree of the body inclination. However, when the support surface is unstable and proprioceptive information from the lower body becomes unreliable, the contribution of vestibular afferentation for determination of the body position becomes critical. In addition, other studies in individuals with vestibular disorders have assessed the role of the vestibular apparatus in postural control (Nashner et al., 1982; Allum and Shepard, 1999). The results of those studies indicate that vestibular afferentation act as an internal reference for proprioceptive and visual inputs. Recently, an extended battery of SOT has been proposed to assess the contribution of vestibular system to postural control in astronauts (Black and Paloski, 1998; Wood et al., 2015). This method allows for the otolith input distortion using dynamic head tilts in the sagittal plane (Jain et al., 2010). It was shown that the complete recovery of postural stability in astronauts was reached within 10–15 days after long-term SF (first 25 expeditions on ISS), and the instability that persists until this time in tests with head tilts is explained by the overestimation of low-frequency otolith signals (Wood et al., 2015). Severity of disequilibrium increases and recovery is prolonged with increasing exposure time to weightlessness (Miller et al., 2018). Return of postural control to baseline occurs ∼4 days after short-duration SF and ∼12 days after long-duration SF (Clément G. R. et al., 2020). Most of investigations connect alterations in sensory-motor system with vestibular system. It is known, that the perception of the horizontal and vertical distances of a visual target changes (Clément G. et al., 2020). Weightlessness alters the input signals of the otoliths and their effect on the pattern and dynamics of changes in the vestibular function (Kornilova et al., 2017; Glukhikh et al., 2022).

Computerized dynamic posturography (CDP) reflects the body center of mass movements and does not take into account the multi-joint coordination, which is the cause of these movements. It has been shown earlier (Horak and Nashner, 1986) that there are two main types of movement strategies that can be used to return the body to equilibrium in a stance position and to keep the feet in place: the ankle strategy, when the center of pressure transition occurs mostly due to the movement around the ankle joints, and the hip strategy which involves movements around the hip joints. Biomechanical models (Alexandrov et al., 2001; Versteeg et al., 2016) have also demonstrated that of possible strategies, the ankle and hip strategies are the most efficient. The hip strategy is engaged during corrections to large postural perturbations, whereas the ankle strategy is used during corrections to minor perturbations. It is known, that hip strategy contribution increases during perturbations in elderly people (Afschrift et al., 2016). Hip strategy involvement also depends on muscle tone (Kaminishi et al., 2020, 2021). Muscle tone registered by microvibrations decreases in microgravity (Gallasch et al., 1998). We assume that this atonic state during long-term SF can lead to increase of hip strategy contribution in postural balance maintenance. Short-term SF (9–16 days) results in hip strategy contribution increase in all SOT conditions except the test with eyes closed and proprioceptive disturbance (Speers et al., 1998), these alterations are associated with changes in sensory processing for motion perception and spatial orientation. Therefore, we hypothesized that transition from the ankle to hip strategy during standing in sensory challenging environment can remain the same in the course of early recovery period (first 3 days after landing). It indicates a growing overload for the postural control to maintain balance, and such a transition could be used as an indicator of sensory deficit following spaceflights.

## 2. Materials and methods

The study involved 33 cosmonauts – crew members of expeditions to the International Space Station, ISS missions 16–66, with an average duration of space flights from 166 to 196 days. The group consisted of 32 men and one woman 46.6 ± 4.9 years old, weight 81.2 ± 8.5 kg, height 177.1 ± 5.3 cm. All the participants provided written informed consent to participate in the study that received ethical approval from the Physiological Section of the Biomedicine Ethics Committee at the Institute of Biomedical Problems of the Russian Academy of Sciences and Human Research Multilateral Review Board (HRMRB).

The studies were conducted twice (on average – 60 and 30 days, L-30 and L-60) before launch, as well as on the 3–4, 7–8, and 10–11 days after SF completion (R + 3, R + 7, and R + 10, respectively). All studies were conducted in the morning, physical activity was excluded before the study.

### 2.1. Study of stabilographic characteristics before and after long-term SF

**FIGURE 1:**
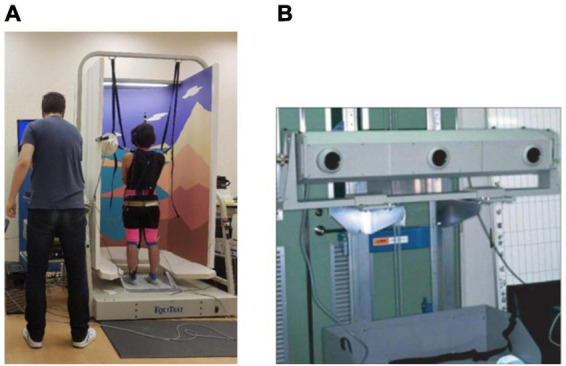
**(A)** Neurocom EquiTest complex. **(B)** Optotrack 3020 motion capture systems.

Subjects were tested according to a modified computerized dynamic posturography (CDP) protocol with the use of EquiTest clinical system (Neurocom, USA) (Figure 1A).

**FIGURE 2:**
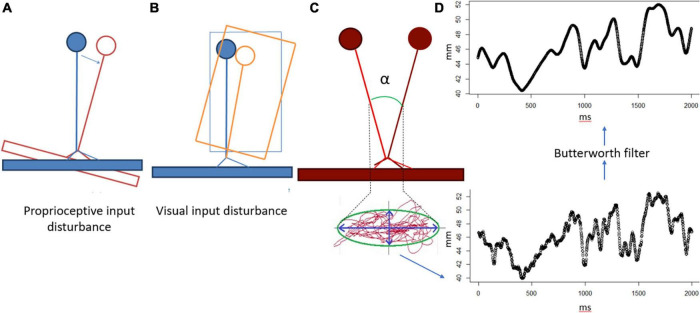
Neurocom data processing. **(A)** Sway-referenced platform for proprioceptive input disturbance. **(B)** Sway-referenced visual surround for visual input disturbance. **(B)** Center of Pressure trace and sway angle. **(D)** Stabilogram alignment with Butterworth filter.

The system consists of sway-referenced stabilometric platform, which rotation axis intersects ankle joints. The visual area is enclosed by sway-referenced visual surround. The frequency of stabilographic signal is 100 Hz. The body inclination is measured in real time by system and platform or surround rotates to make the proprioceptive or visual input unreliable (Figures 2A, B). Such conditions provide distortion or elimination (in the case of vision) of different afferent inputs: proprioceptive - with a sway referenced platform, visual - with a sway referenced screen, vestibular - with head tilts. This method of testing the contributions of various sensory systems in vertical stance control was proposed by Nashner (1971), who developed the EquiTest research complex, which development described in Black and Paloski (1998). When processing the data, the complex dimensionless vertical stability indicator Equilibrium Score (EqScore) was analyzed, which was calculated by the formula:

EqScore = (1 – α/12,5°) × 100,

where α is the maximum angle of the center of mass fluctuation for the entire recording period (Figure 2C). In a normal population, this indicator is 12.5°. The displacement of the center of gravity was calculated by filtering the sagittal stabilogram with a Butterworth high-frequency filter with a cutoff frequency of 0.85 Hz (Figure 2D). The detailed description is in Wood et al. (2015).

**FIGURE 3:**
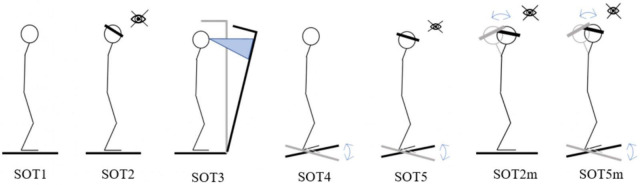
Sensory organization tests.

During the assessment subjects wore headphones in order to block ambient noises and unwanted auditory cues as well as to play a pacing sound for the tests involving head movements. A white noise was played through the headphones to muffle ambient sounds. The pacing sound for modified tests with head tilts was represented by a continuous tone frequency modulated by a 0.33 Hz sine wave. Each experimental session consisted of seven tests with different conditions. Five tests are standard for studies using the EquiTest Neurocom complex (Figure 3):

- SOT1 – vertical stance with eyes open;

- SOT2 – vertical stance with eyes closed;

- SOT3 – vertical stance with eyes open and a sway referenced visual surrounding;

- SOT4 – a stand with open eyes and a sway referenced support surface;

- SOT5 – vertical stance with eyes closed and a sway referenced support surface.

The modified tests proposed by Jain et al. (2010) were also included in the program:

- SOT2m – vertical stance with eyes closed while performing dynamic head tilts in the sagittal plane with a frequency of 0.33 Hz and an amplitude of 40°;

- SOT5m – vertical stance with eyes closed, a moving platform (as in SOT5) and while performing dynamic head tilts.

When analyzing the data, two-factor analysis of variance (ANOVA) with Bonferroni correction was used for normal distribution, the graphs are presented as averages and standard deviations.

### 2.2. Study of kinematic characteristics

**FIGURE 4:**
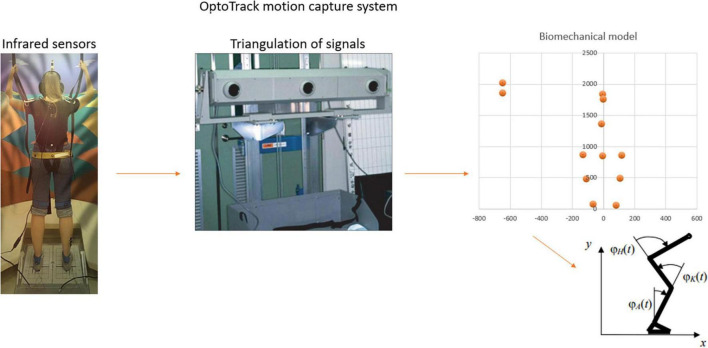
Kinematic characteristics registration and processing.

The cosmonauts were equipped with a system of infrared sensors (NDI OptoTrack, USA). Basing on the data of these sensors the body scheme was built for each subject using Rstudio software. Sensors were attached to the right heel, right popliteal cavity, right hip and headphones (to control the amplitude and frequency of head tilts). Angles in the ankle, knee and hip joints were calculated (Figure 4). The average velocities of the angle fluctuations and the Random Mean Square (RMS) of these fluctuations were analyzed.

Sensorimotor system applies unlimited number of ankle-hip strategy combinations (Horak and Nashner, 1986; Horak, 2006). We determined the change in the contribution of the hip/ankle strategy by calculating the ratio between the ratios of the RMS of fluctuations in the hip and ankle joints before and after the flight:

HASC = (HR_*R+*3_/AR_*R+*3_)/(HR_*L–*60_/AR_*L–*60_)

where HASC – hip/ankle strategy contribution changing, HR – hip angle RMS, AR – ankle angle RMS.

Due to the large variability of the data, statistical analysis was carried out using the non-parametric Wilcoxon test, which allows comparing samples with an abnormal distribution. Graphically, the data are presented as medians with an interquartile range.

We used standard level for medical investigations – *p* < 0.05. We used open source software for data science RStudio for analysis of device signals and statistical processing. We used GraphPad Prizm 8 for making figures.

## 3. Results

### 3.1. Stabilometric characteristics after long-term SF

**FIGURE 5:**
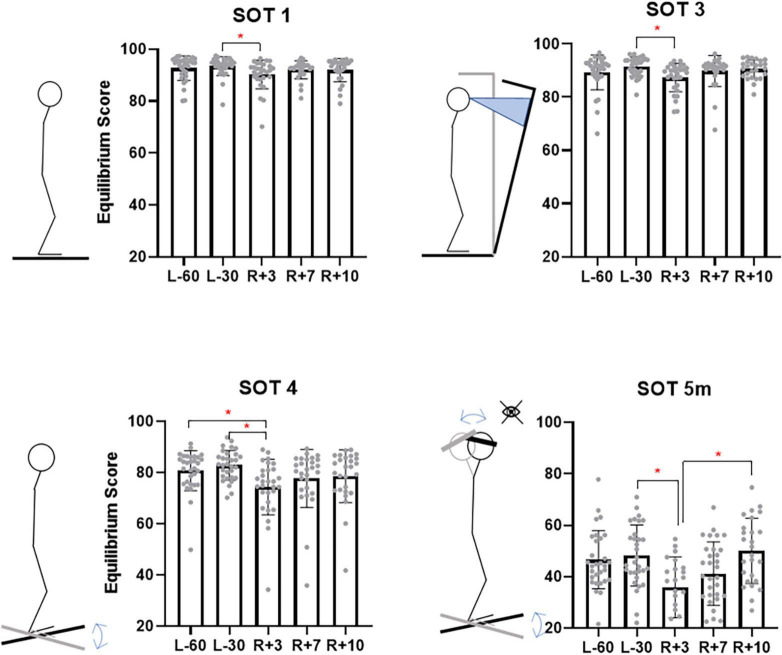
Sensory organization tests with significant changes between pre- and postflight Equilibrium Score. L-60 – 60 days before launch, L-30 – 30 days before launch, R + 3 – 3rd day of recovery period after landing, R + 7 – 7th day after landing, R + 10 – 10th day after landing.

In the simplest condition – SOT1 – on a stable platform and with eyes open – there was a significant decrease in EqScore values on the 3rd day after landing (on average from 93.5 to 90.3 points, *p* = 0.017). Significant changes were also observed in the SOT3 tests - with eyes open and a moving visual environment (from 91.6 to 87.3 points, *p* = 0.0029), in the SOT4 test - with eyes open and a sway referenced support surface (from 82.9 to 74.4 points, *p* = 0.0027), in the SOT5m test - with eyes closed, a sway referenced support surface and head tilts – from 48.2 to 35.9, *p* = 0.016 (Figure 5).

**FIGURE 6:**
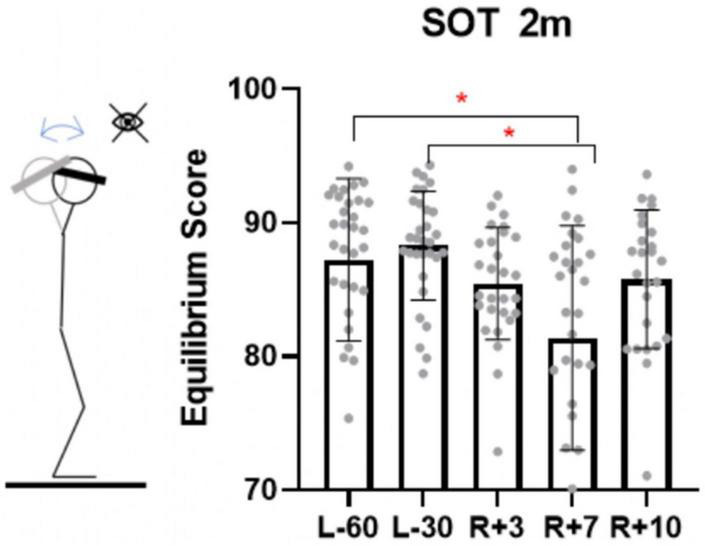
Sensory organization test with eyes closed, fixed platform and head tilting (proprioceptive testing). L-60 – 60 days before launch, L-30 – 30 days before launch, R + 3 – 3rd day of recovery period after landing, R + 7 – 7th day after landing, R + 10 – 10th day after landing.

It was found that in one of the conditions – in the test with eyes closed, fixed support surface and head tilts (SOT2m) – no significant changes were observed on the 3rd day after landing, but they were detected on the 7th day (Figure 6, *p* = 0.006 for comparison of L-60 and R + 3 and *p* = 0.0019 for comparison L-30 and R + 3).

### 3.2. The influence of space flight factors on the kinematic characteristics of vertical stance in cosmonauts

**FIGURE 7:**
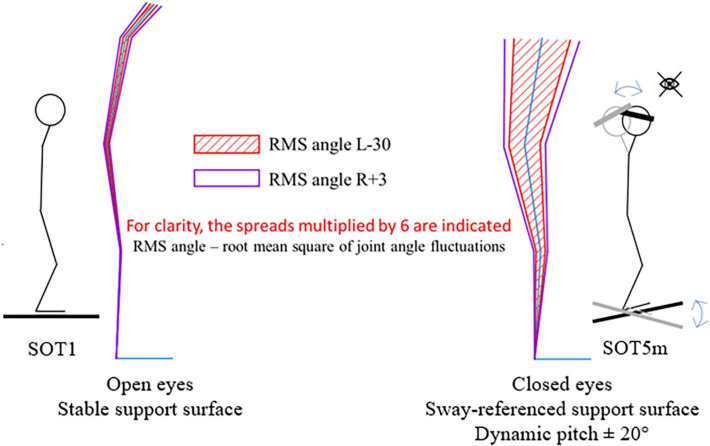
Increase of angles RMS between SOT1 and SOT5.

Under conditions of absent visual feedback, distorted proprioceptive and vestibular input (SOT5m) we saw greater root mean square of joint angles, than in other tests and on day R + 3 (0.17° in SOT1 and 0.26° in SOT5m in ankle, *p* < 0.001, 0.12° in SOT1 and 0.55° in SOT5m, *p* < 0.001) (Figure 7).

Vertical balance maintenance can be represented as stabilization of a three-link inverted pendulum. There are three main strategies for maintaining balance: hip, knee, and ankle ones (Alexandrov et al., 2001). These strategies are independent by central control. The ankle strategy is much more inertial than the hip one, and is used for slow movements of the center of gravity, the hip strategy – for fast movements (Horak and Nashner, 1986; Afschrift et al., 2016). The knee strategy makes a small contribution into vertical balance control (Kuo et al., 1998; Alexandrov et al., 2001), so we did not consider it. The movements of the center of gravity and, consequently, the central of pressure, are linked to the joints’ angles. So, we focused our attention on the changes in those equilibrium tests in which significant changes in the stabilometric characteristics were detected.

**FIGURE 8:**
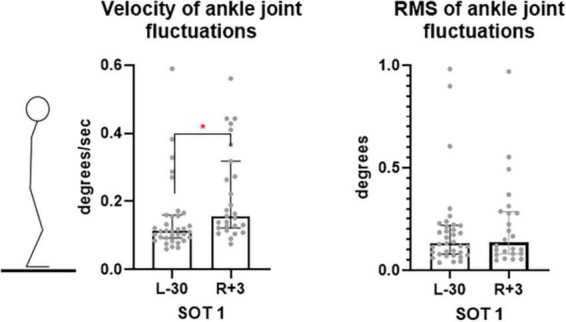
Ankle joint fluctuation velocity is SOT1 test. L-30 – 30 days before launch, R + 3 – 3rd day of recovery period after landing.

In the SOT1 test – eyes open, fixed support surface – significant changes in the angular velocity in the ankle joint were detected (from 0.13° to 0.17° *p* = 0.014), while no changes were observed in the hip joint. Thus, the vertical balance maintenance in this case was provided by an ankle strategy typical for vertical stance under normal conditions (Figure 8).

**FIGURE 9:**
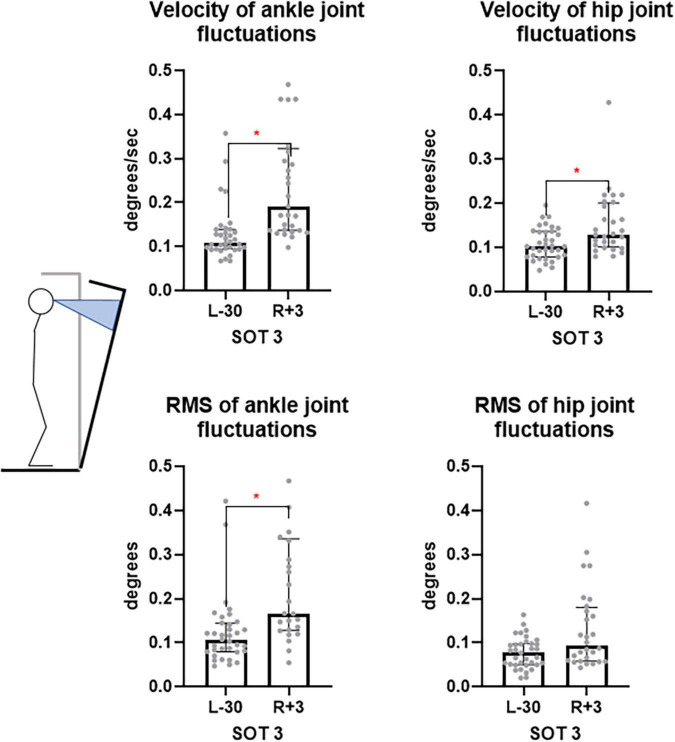
Ankle and hip joints fluctuation velocity and RMS is SOT3 test. L-30 – 30 days before launch, R + 3 – 3rd day of recovery period after landing.

In the SOT3 test – eyes open, moving visual environment– significant changes in angular velocity and RMS of the ankle joint position (from 0.11° to 0.19°, *p* < 0.0001) and a significant increase in the angular velocity in the hip joint (from 0.10° to 0.13°, *p* = 0.03) were detected, however, no significant increase in the RMS of hip joint position was registered (Figure 9). Since the increase in the angular velocity in this case is not accompanied by increase of angular RMS, we assume that these changes occur within the frame of the ankle strategy.

**FIGURE 10:**
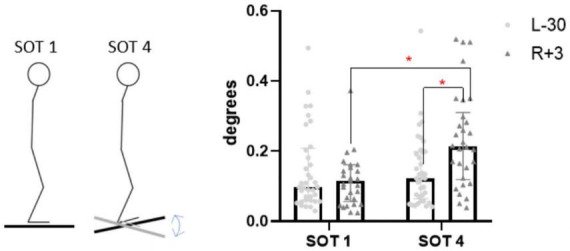
Hip joints fluctuation RMS in SOT1 and SOT4 tests. L-30 – 30 days before launch, R + 3 – 3rd day of recovery period after landing.

In the SOT4 test – eyes open, sway referenced support surface – on the 3rd day after the completion of SF, significant changes in the RMS of the hip joint position were detected (from 0.12° to 0.21°, *p* = 0.03), as well as a significant difference from SOT1 hip joint RMS, conducted in the same post-flight session (*p* = 0.02) (Figure 10). These changes suggest the recruitment of a hip strategy for maintaining vertical balance.

**FIGURE 11:**
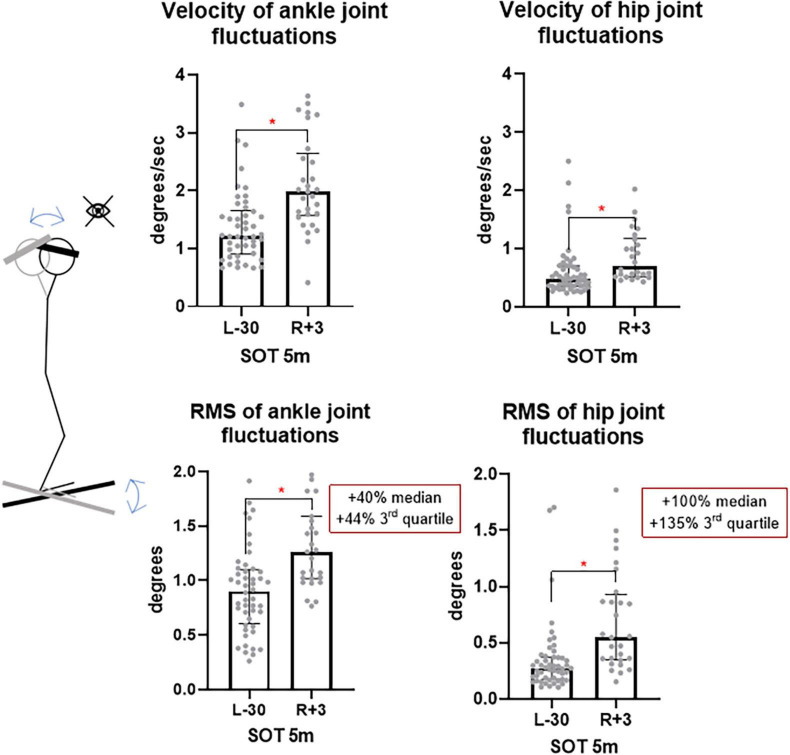
Ankle and hip joints fluctuation velocity and RMS is SOT5m test. L-30 – 30 days before launch, R + 3 – 3rd day of recovery period after landing.

**FIGURE 12:**
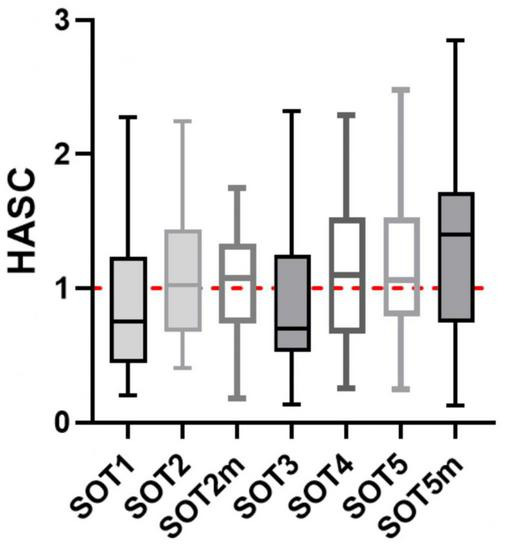
Hip/ankle strategy contribution (HASC) changes between L-30 and R + 3. In ordinate: ratio of hip/ankle strategy involvement indicator (after flight/before flight).

In the SOT5m test – eyes closed, sway referenced support surface and head tilts – significant changes in the angular velocity and RMS were found both in the ankle (from 0.90° to 1.26°, *p* < 0.001) and in the hip joints (from 0.27° to 0.55°, *p* < 0.001), which corresponds to 40% an increase in the median value and a 44% increase in the 3rd quartile in the ankle joint, a 100% increase in the median and a 135% increase in the 3rd quartile in the hip joint (Figure 11), which suggests an increase in the contribution of the hip strategy to maintaining balance (Kaminishi et al., 2021). This is confirmed by the hip/ankle strategy contribution changes (Figure 12), where the median value of SOT5m is the biggest one (1.4). The values less than 1 for SOT1 (0.75) and SOT3 (0.7) confirm the ankle strategy involvement to postural changes in this test.

**FIGURE 13:**
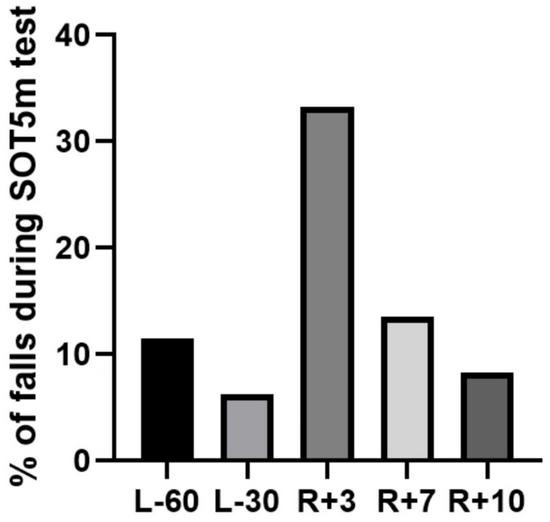
Percent of falls in SOT5m test. L-60 – 60 days before launch, L-30 – 30 days before launch, R + 3 – 3rd day of recovery period after landing, R + 7 – 7th day after landing, R + 10 – 10th day after landing.

There were falls (steps during test to avoid losing the balance) during SOT5m performance. Other tests were performed clearly. The majority of falls were in R + 3 session (33.3% from summary number of all cosmonauts attempts), but there were also falls in other experiment sessions (Figure 13). The decrease from 11.5 to 6.25% from L-60 to R + 3 could indicate the learning effect between 1st and 2nd experiment sessions and decrease from R + 3 to R + 10 – about recovery processes.

**FIGURE 14:**
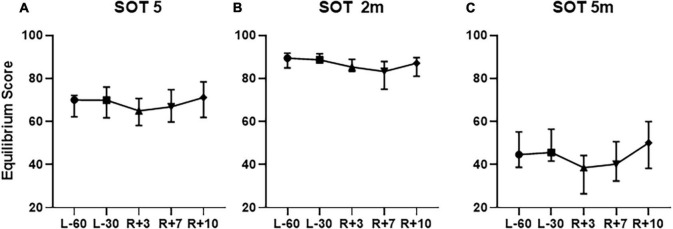
Median Equilibrium Score in **(A)** SOT5, **(B)** SOT2m, **(C)** SOT5m tests. L-60 – 60 days before launch, L-30 – 30 days before launch, R + 3 – 3rd day of recovery period after landing, R + 7 – 7th day after landing, R + 10 – 10th day after landing.

The comparison between Equilibrium Score in our study and in Wood et al. (2015) revealed that changes after SF are similar except the SOT5, 2 m, 5 m conditions. We calculated the median of these tests parameters to compare them with recovery curves in Wood et al. (2015). It was found that cosmonauts in our study have less Equilibrium score in SOT5 (65 against 75 on R + 3, Figure 14A) and greater - in SOT2m and 5 m (85 against 75 on R + 3, Figure 14B, 38 against 25 on R + 3, Figure 14C).

## 4. Discussion

The results allowed us to quantify changes in postural control after long-term SF. Tests that did not directly assess the contribution of vestibular system (i.e., SOT1-SOT5), revealed recovery of postural control to the baseline values by R + 7. However, those tests that required increased engagement of vestibular system (i.e., SOT5m) indicated the recovery only on R + 10, which is in accordance to previous studies (Wood et al., 2015). Further analysis of the SOT5m test results demonstrated a progressive EqScore recovery: there was a positive dynamic of the score on R + 10 relative to R + 3 session. This observation once again confirms the significant effects of SF on the vestibular system and demonstrates that post-flight long-term recovery of balance is associated with the function of this sensory system.

Comparison of our results with recovery curves in SOT5, 2 m and 5 m from Wood et al. (2015) revealed the difference between two experiments in spite of exactly the same device and experimental battery used. Five of 25 ISS expeditions used Shuttle instead of Soyuz, seven expeditions used both types of ships. It is known, that Shuttle provides significantly less g-loads during landing, so we should expect better postural stability, but this was observed only in SOT5 test.

One of the first training procedures for cosmonauts was the vestibular training (CVT, rotating chair with environment fixed to rotating axis). Previous studies of the effects of CVT (Clément et al., 2001) revealed the decrease of motion sickness severity and signs of vestibular adaptation. We can suggest that difference in EqScore values in two mentioned studies could be explained by the difference in vestibular training protocols used before SF.

The other possible cause of the difference in postural stability after landing could be the physical training protocols used by the crew members in the course of SF. Both cosmonauts and astronauts perform the program of physical training on ISS, including treadmill running, cycling and resistive training. The benefit of physical training in microgravity is the well-known fact (Kozlovskaya et al., 2015), but we didn’t find any mentions on comparative studies between RSA and NASA/ESA training programs.

The alterations in contribution of proprioception to postural control (SOT2m, test with eyes closed, vestibular disturbance and without proprioceptive disturbance) were not registered. Unfortunately, in our study, we had a possibility to perform the first postflight tests only on the 3rd day after landing. At the same time, Wood et al. (2015) observed the 25% decrease of EqScore in this test on 1st day after landing. Perhaps these alterations could disappear by the 3rd day after landing.

It has been previously demonstrated that patients with impaired vestibular function have a reduced or even absent ability to titrate the stretch reflex response in the lower leg muscles to displacements and rotations of the supporting surface (Nashner, 1976). Other studies (Reschke et al., 2009; Miller et al., 2018) report about changes in proprioceptive input after SF and its ground-based model - head-down tilt bed rest (HDBR). However, these conclusions were made basing on the stretch reflex examination (Reschke et al., 2009) and locomotor analysis (Miller et al., 2018). The CDP was also used in these studies, but no significant changes were revealed in SOT1-5 after 42–63 days of HDBR (Reschke et al., 2009). Changes in SOT5m, revealed after 70-day HDBR (Miller et al., 2018), reflect the vestibular input changes, but not proprioceptive one. It was shown that SF is followed by the alterations of sensory processing of vestibular input, but appropriate visual and proprioceptive information could blunt the effect of this deficit. SOT5m was developed for analysis of this deficit, not for the proprioceptive system assessment (Jain et al., 2010).

Our study has also revealed changes in the postural strategies that cosmonauts used after SF under conditions when proprioceptive or vestibular inputs were distorted (i.e., SOT4, SOT5m). Other studies (Speers et al., 1998) also noted an increased contribution of the hip strategy in similar tests within a few hours after landing following short-term SF (i.e., 7 to 16 days). In our study we have information only about recovery period from 3 to 10 days after landing. The changes in postural strategies pattern are observed on R + 3, but only in tests, where vestibular input was critical. The most pronounced changes toward increase of the hip strategy contribution were revealed in the SOT5m test, when visual feedback is absent, while proprioceptive and vestibular inputs are distorted. We registered a greater usage of hip strategy in posture maintenance compared to ankle strategy usage in this test. In SOT4, the least complicated test with proprioceptive input distortion, we also observed increase of hip strategy contribution after SF. Recent studies report about the updating of a cerebellum-based internal model of the sensory consequences of gravity and about the re-weighting of extra-vestibular information (Carriot et al., 2021). Previous studies also attributed the increase of hip strategy contribution to the alterations in central processing of sensory information (Speers et al., 1998).

The greatest changes in the postural control were observed in the postflight tests introducing the greatest challenge for the vestibular system. This is not surprising given that vestibular neglect develops within the first days of SF (Kornilova et al., 2017). As it was shown in studies of the vestibular-ocular reflex after SF (Glukhikh et al., 2022) vestibular system recovery takes up to 9 days after landing, which can affect the recovery of the vestibular contribution in postural control.

## 5. Conclusion

1. The changes occurring in the vestibular system may play the leading role in reducing postural stability after long-term space flight.
2. The changes in postural strategy patterns remain up to 3rd day after landing, which could be the evidence of long duration alterations in central processing of spatial information.
3. Proprioceptive input processing recovers much earlier, than vestibular one.

## Data availability statement

The raw data supporting the conclusions of this article will be made available by the authors, without undue reservation.

## Ethics statement

The studies involving human participants were reviewed and approved by the Biomedicine Ethics Committee at the Institute of Biomedical Problems of the Russian Academy of Sciences and Human Research Multilateral Review Board (HRMRB). The patients/participants provided their written informed consent to participate in this study.

## Author contributions

DS, VK, and ET designed the study. NS and VK conducted the research. NS processed the data and drafted the manuscript. VK and DS revised the manuscript. ET contributed in the global revision of the manuscript and was a supervisor of the experiment. All authors interpreted the data and have read and approved the final submitted manuscript.
